# A study of the mechanism of lncRNA-CR594175 in regulating proliferation and invasion of hepatocellular carcinoma cells in vivo and in vitro

**DOI:** 10.1186/s13027-020-00321-8

**Published:** 2020-09-22

**Authors:** Quan Liu, Xuxu Yu, Minjie Yang, Xiangke Li, Xuejia Zhai, Yujin Lian, Zhong Chen, Qingxia Fan, Lijie Song, Wencai Li

**Affiliations:** 1 Department of Emergency, The Fifth Affiliated Hospital of Zhengzhou University, Zhengzhou, 450052 China; 2 Department of Oncology, The First Affiliated Hospital of Zhengzhou University, Zhengzhou, 450052 China; 3 Department of Pathology, The First Affiliated Hospital of Zhengzhou University, Zhengzhou, 450052 China

**Keywords:** lncRNA, lncRNA-CR594175, Hepatocellular carcinoma, Hsa-miR142-3p, HepG2

## Abstract

**Background:**

Hepatocellular carcinoma (HCC) is one of the cancers of highest incidence and mortality worldwide. The proliferation and invasion of tumor cells are the main reason for poor prognosis after HCC surgery. Long non-coding RNA (lncRNA) has been shown to play a key role in the progression of HCC. LncRNA-CR594175 is one of the highly expressed lncRNAs in HCC tumors and their metastatic tumors that we have obtained by the High-throughput screening method.

**Methods:**

To elucidate the role of lncRNA-CR594175 in regulating the proliferation and invasion of human hepatoma cell line, HepG2, we operated through lncRNA-CR594175 silencing to inhibit the progression of HCC, either through in vitro or in vivo experiments.

**Results:**

We found that lncRNA-CR594175 was lower in adjacent non-cancerous tissues than in primary HCC, and was lower in primary HCC than in its metastasis. Silencing of lncRNA-CR594175 inhibited the proliferation and invasion of HepG2 cells and growth of subcutaneous tumors. The results revealed that lncRNA-CR594175, as a RNA sponge, broke the negative regulation of hsa-miR-142-3p on Catenin, beta-1 (CTNNB1), and once lncRNA-CR594175 was silenced, the hsa-miR142-3p regained its negative regulation on CTNNB1 which can promote HCC progression by activating the wnt pathway.

**Conclusions:**

Our present study demonstrated for the first time that lncRNA-CR594175 silencing suppressed proliferation and invasion of HCC cells in vivo and in vitro by restoring the negative regulation of hsa-miR-142-3p on CTNNB1, laying a solid theoretical base for using lncRNA-CR594175 as genetic target therapy for HCC and offering a reasonable explanation for inactivation of miRNA in different tumors or in the tumor at different stages.

## Background

Hepatocellular carcinoma (HCC) is one of the cancers of highest incidence and mortality worldwide [1], featuring a low complete resection rate and a high postoperative recurrence rate [2, 3], which is mainly driven by high invasiveness and intrahepatic and/or extrahepatic metastasis [4]. Therefore, the study of the mechanisms involved in HCC cell proliferation and invasion may be of great significance to identify prognostic markers in HCC patients. At present, surgery combined with pre- and post-operative chemotherapy is the mainstay of treatment for HCC but this traditional therapeutic method doesn’t work for postoperative recurrence and metastasis. So, in recent years, searching for new therapeutic targets for HCC treatment never stopped.

Both microRNAs (miRNAs) and lncRNAs play important roles in regulating cellular processes [5–7]. Going through all lncRNAs identified by the screening and sequencing of the transcriptome, lncRNA-CR594175 was the lncRNA most differentially expressed among adjacent non-cancerous tissues, HCC and metastases. The expression of lncRNA-CR594175 increases from adjacent non-cancerous, to primary HCC, to metastasis tissues, which suggests that lncRNA-CR594175 may be involved in proliferation and invasion of HCC. According to our screening data, CTNNB1 was highly correlated with the process of HCC development.

The pathogenesis of HCC is complex and Wnt/*CTNNB1* signaling pathway plays a central role in the hepatocarcinogenesis. According to medical basic research, Wnt/*CTNNB1* signaling pathway can affect the process of HCC mainly by expression regulation of downstream genes and proteins. The increasing expression of CTNNB1 leads to cell proliferation according to the Wnt/*CTNNB1* signaling pathway. It makes CTNNB1 a critical gene in the research of HCC regulation pathways. The inhibitory effects of Wnt signaling pathway by nonsteroidal anti-inflammatory drugs and valproic acid has been used as adjuvant for HCC therapy [8], therefore, *CTNNB1* inhibitors become the new study direction in preventing precancerous lesions such as hepatitis and liver cirrhosis from deteriorating. It has already been verified that R-Etodolac, as the inhibitor of *CTNNB1*, can suppress proliferation of HCC cell lines HepG2 and Hep3B effectively [9]. In addition to this, a variety of miRNAs has been confirmed to have an influence on the process of HCC through the regulation of Wnt/*CTNNB1* signaling pathway [10–12] and the targets are different. It’s uncommon for miRNA to involve Wnt signaling pathway targeting *CTNNB1* until now, but our study revealed that miRNA hsa-miR-142-3p has a negative effect on the HCC progression by affecting the *CTNNB1* pathway. The data indicated an increase in the expression level of hsa-miR142-3p from HCC metastasis to primary lesion and then to adjacent non-cancerous tissues, but it’s puzzling that the expression of CTNNB1 protein remains stable. So, it’s crucial for the theoretical research and clinical treatment to find the reason why antineoplastic miRNAs such as hsa-miR-142-3p are inactivated in the progression of HCC. The study on the interaction between miRNAs and lncRNAs will revolutionize our knowledge about cell structural network and regulatory network, and bring in immeasurable scientific and clinical value.

## Materials and methods

### Cell culture

Human hepatoma cell line, HepG2, obtained from the Cell Bank of the Chinese Academy of Sciences (Shanghai, China), were maintained in RPMI-1640 (Invitrogen, CA, USA) supplemented with 10% Fetal bovine calf serum (FBS, Invitrogen, CA, USA). 293TN cells, purchased from ATCC (MD, USA), were maintained in Dulbecco minimum essential medium (DMEM, Invitrogen CA, USA) supplemented with 10% FBS. All these adherent cells were passaged by 0.25% trypsin digestion (Invitrogen CA, USA) and incubated in an atmosphere of 5% CO_2_ at 37 °C.

### Assessment of lncRNA-CR594175, hsa-miR142-3p, CTNNB1 protein and Wnt pathway related protein expression levels in HCC tumors and their metastasis

Adjacent non-cancerous tissues, HCC, the metastatic from 24 patients (diagnosed in the First Affiliated Hospital of Zhengzhou University and detailed patient information was shown in Table 1) were collected, followed by total RNA extraction and Quantitative Real-time PCR (RT-qPCR) for measurement of lncRNA-CR594175 and hsa-miR142-3p level and total proteins were extracted and used for CTNNB1 and Wnt pathway related proteins (E-cadherin, C-myc, CyclinD1 and MMP-9) detection by western blotting.

**Table 1:** Clinicopathological features of 24 patients with metastatic HCC

Number	Gender	Age	TNM stage	Metastasis Site
1	F	55	T2N1M1	Lung
2	M	62	T3N1M1	Lung
3	F	61	T2NXM1	Stomach
4	F	55	T2N0M1	Head
5	F	58	T3NXM1	Colon
6	M	66	T3N0M1	Lung
7	M	73	T2N1M1	Stomach
8	F	55	T3N1M1	Colon
9	M	42	T2N1M1	Stomach
10	F	58	T3N0M1	Lung
11	M	51	T3N1M1	Stomach
12	M	44	T2N0M1	Stomach
13	F	53	T3NXM1	Lung
14	F	66	T3N1M1	Colon
15	M	62	T3N0M1	Stomach
16	M	60	T2NXM1	Lung
17	F	52	T3N1M1	Lung
18	F	47	T3N0M1	Lung
19	M	52	T2N0M1	Bone
20	F	58	T2N0M1	Stomach
21	M	69	T3NXM1	Lung
22	F	70	T2NXM1	Lung
23	F	42	T4N1M1	Colon
24	M	53	T3N1M1	Bone

M, male; F, female; TNM, tumor node metastasis.. Clinical stage category were ruled by 7th edition of TNM

There are 24 pairs HCC tumors, their metastasis and adjacent normal tissues obtained from patients in the First Affiliated Hospital of Zhengzhou University between January 2013 and December 2017. Clinical stage category was ruled by 7th edition of TNM

### Lentivirus packaging

A siRNA sequence complementarily binding to lncRNA-CR594175 was chosen. The target sequences of siRNA (5′-GAATCCTCGGAGACAGCAG-3′) are homologous to lncRNA-CR594175.The oligonucleotide templates of these shRNAs were chemically synthesized and cloned into the linear pSIH1-H1-copGFP shRNA Vector (System Biosciences, CA, USA) which was obtained through digestion by BamH I and EcoR I (Takara, Dalian, China) and purification by agarose gel electrophoresis. An invalid siRNA sequence (5′- AATCGTCGAGGGCCAGACA-3′) was used as a negative control (NC). Sequencing was used to confirm the vectors constructed (pSIH1-shRNA-CR594175 and pSIH1-NC). The CDS sequence of human CTNNB1 (NM_001904.3) was amplified by using the primers 5′-GGAATTCGCCACCATGGCTACTCAAGCTGATTTG-3′ and 5′-CGGGATCC TTACAGGTCAGTATCAAACC-3′, which contain an EcoRI cutting site and Kozak sequence and a BamhI cutting site, respectively, with the cDNA prepared by reverse transcription of RNA isolated from 293TN cells. The PCR product was digested and cloned into pcDH1-CMV lentiviral expressing vector; the recombinant vector was named pcDH1-CTNNB1. The products of the vectors were confirmed by DNA sequencing. Endotoxin free DNA was prepared in all cases.

One day before transfection, 293TN cells were seeded into 10-cm dishes (Corning, NY, USA). 2 μg of each pSIH1-shRNA-CR594175 vector or pSIH1-NC and 10 μg pPACK Packaging Plasmid Mix (System Biosciences) were co-transfected using Lipofectamine 2000(Invitrogen) in accordance with the manufacturer’s protocol. The medium was replaced with DMEM plus 1% FBS. Forty eight hours later, the supernatant was harvested and then cleared by centrifugation at 5000×g at 4 °C for 5 min, and passed through a 0.45 μm PVDF membrane (Millipore, MI, USA). The titer of virus was determined by gradient dilution. The packaged lentiviruses were named as Lv-shRNA-CR594175 and Lv-NC. Recombinant lentivirus Lv-CTNNB1 and Lv-miR142-3p were packaged by following the same protocol.

### Genetic intervention through a lentiviral approach

Cells were divided into four groups: a control group, Lv-NC group (infected with Lv-NC), Lv-shRNA-CR594175 group (infected with Lv-shRNA-CR594175) and Lv-CTNNB1 group (infected with Lv- CTNNB1). HepG2 in logarithmic phase growth were seeded to 6-well plates at 5 × 10^5^ cells/well. One day later, viral solution was added at a multiplicity of infection (MOI) of 10. The infection efficiency was evaluated by observing and analyzing the fluorescent mark 72 h after infection. Total RNA and protein were isolated from the cells and subjected to RT-qPCR and western blotting for lncRNA-CR594175 and CTNNB1 protein.

### Luciferase experiment

Total RNA was extracted from HepG2, reverse-transcribed into cDNA, and 2 μl of the reaction product subsequently used as a template for PCR. Primers targeting the 3′-UTR of the CTNNB1 gene were designed such that flanking XbaI restriction sites were introduced into the 127 bp (base-pair) PCR product containing the 5′-AACACTA-3′ hsa-miR-142-3p target site. The forward and reverse primer sequences were 5′- GCTCTAGATTAAGAATTGAGTAATGG-3′ and 5′-GCTCTAGA ACTAATTGGACCATTTTC-3′, respectively. PCR reaction conditions were as follows: 35 cycles of a 94 °C denaturing step for 30 s, a 55 °C annealing step for 30 s, and a 72 °C elongation step for 10 s. The PCR product was digested with XbaI (Takara) and cloned into the pGL3-promoter luciferase reporter vector (Promega, MI, USA) to generate the vector pGL3-wt-CTNNB1. The hsa-miR142-3p target site in the pGL3-WT-CTNNB1 vector was mutated from 5′- AACACTA − 3′ to 5′- CATAACA − 3′ to construct the mutated reporter vector, pGL3-mt-CTNNB1. The products of all cloning and mutagenesis reactions were confirmed by DNA sequencing. Endotoxin free DNA was prepared in all cases. The hsa-miR142-3p mimic(5′-UGUAGUGUUUCCUACUUUAUGGAtt-3′), the hsa-miR142-3p inhibitor (5′- UCCAUAAAGUAGGAAACACUACAtt-3), and negative control miRNA (NC,5′- UGUAGUGUUUCCUACUUUAUGGAtt-3′) were all chemically synthesized (Invitrogen).

We used Targetscan (http://www.targetscan.org/) to predict whether a hsa-miR142-3p binding site exists within the 3′-UTR of human CTNNB1 mRNA. The results showed that a seven-base hsa-miR142-3p seed sequence is present in the 3′-UTR of CTNNB1 mRNA. The same tool was used to predict the binding sites of hsa-miR142-3p on lncRNA-CR594175.A suspension of 293TN cells in logarithmic phase growth was prepared and the number of viable cells counted using a hemocytometer in conjunction with trypan blue staining. The cells were seeded into six-well plates at a concentration of 2 × 10^5^ cells per well and maintained in Dulbecco’s Modified Eagle’s medium supplemented with 10% FBS at 37 °C for 24 h in a 5% CO_2_ atmosphere. The transfection of plasmid DNA and RNA was performed using Lipofectamine 2000 (Invitrogen). Transfection of cells with pRL-TK (100 ng) served as a reference for luciferase detection. Luciferase activity was measured using the dual luciferase reporter assay system (Promega) 48 h after transfection. The experiment to observe the effect of lncRNA-CR594175 depletion on the inhibition of luciferase by hsa-miR142-3p mimics was carried out in 293TN and HepG2 cells; the plasmid transfection and luciferase activity assay were the same as used in the validation of target sites of hsa-miR142-3p.

### Cellular proliferation assay

HepG2 cells were divided into seven groups: a control group, Lv-NC group, Lv-shRNA-CR594175 group, Lv-miR142-3p group, Lv-shRNA-CR594175 and Lv-miR142-3p group, Lv-CTNNB1 group, and Lv-shRNA-CR594175 and Lv-CTNNB1 group. Fourty-eight hours after infection, HepG2 cells groups were trypsinized, and seeded into 96-well plates at a density of 1 × 10^4^ cells per well. The cells were cultured under normal conditions and cell viability was examined using CCK-8 at 24-, 48-, and 72-h time points. Briefly, 10 uL CCK-8 solution (Dojindo, Japan) was added, and the cells then cultured under normal conditions for an additional 4 h before measurement of absorbance at 490 nm.

### Cell invasion assay

Cell invasion experiments were performed using the QCMTM 24-well Fluorimetric Cell Invasion Assay kit (Chemicon, International, MI, USA) according to the manufacturer′s instructions. The kit uses an insert polycarbonate membrane with an 8-μm pore size. The insert was coated with a thin layer of EC Matrix that occluded the membrane pores and blocked migration of non-invasive cells. Culture medium (500 μl) supplemented with 10% FBS was used as chemoattractant. Cells that migrated and invaded the underside of the membrane were fixed in 4% paraformaldehyde. The invading cells were stained by Calcein-AM, and the number was then determined by fluorescence and reported as relative fluorescence units (RFUs).

### Effect of lncRNA-CR594175 silencing on the protein levels of CTNNB1, E-cadherin, C-myc, CyclinD1 and MMP-9

HepG2 cells were divided into three groups: a control group, Lv-NC group and Lv-shRNA-CR594175. Cells in logarithmic phase were seeded to 6-well plates at 5 × 10^5^ cells/well. One day later, viral solution was added and the infection efficiency was evaluated by observing and analyzing the fluorescent mark 72 h after infection. Proteins were isolated and subjected to western blotting for CTNNB1, E-cadherin, C-myc, CyclinD1 and MMP-9 protein, respectively.

### RT-qPCR

Total RNA was isolated with Trizol Reagent (Invitrogen) according to the manufacturer’s instruction and reversely transcribed into cDNA using M-MLV Reverse Transcriptase (Takara, Japan) and oligo (dT)18 primer (Takara, Dalian, China). The following specific primers were used in RT-qPCR of human lncRNA-CR594175 and β-actin: lncRNA-CR594175: forward 5′-TTATGACACATGCCACAACA-3′ and reverse 5′-GGTACCTGTTATAAGTAGAATCA-3′; β-actin: forward5’-CCTGTACGCCAACACAGTGC-3′ and reverse 5′-ATACTCCTGCTTGCTGATCC-3′. The lengths of amplified products were 109 bp and 211 bp, respectively. RT-qPCR was performed using SYBR Premix Ex Taq kit and TP800 System (Takara). cDNA from 200 ng total RNA was used as the template. The PCR reaction was carried out under the following conditions: 40 cycles of denaturation at 95 °C for 10 s, annealing at 60 °C for 20 s and extension at 72 °C for 20 s. The relative levels of mRNA and hsa-miR-142-3p were normalized using the 2^-△△Ct^ method by using β-actin and U6 as the references. The PCR primers for hsa-miR142-3p or U6 were as follows: hsa-miR142-3p: forward: 5′- TGTAGTGTTTCCTACTTTATGGA-3′ and reverse: 5′-GTCGTATCCAGTGCGTGTCGTG-3′; U6: forward:5′-GTGCTCGCTTCGGCAGCACAT-3′ and reverse: 5′-TACCTTGCGAAGTGCTTAAAC-3′.

### Western blotting

The total protein was extracted from the cells and tissues by using mammalian protein extraction reagent (Pierce, IL, USA). Equal amounts of protein (25 μg per lane) estimated by a bicinchoninic acid (BCA) protein assay kit (Pierce) were loaded onto (11%) SDS-PAGE gels and transferred onto nitrocellulose membranes. The blots were probed with a monoclonal antibody against human CTNNB1 (1:400), E-cadherin (1:200), C-myc (1:300), CyclinD1 (1:400), MMP-9 (1:250) and β-actin (1:1000) (Santa Cruz, USA), followed by the secondary HRP-conjugated anti-mouse/rabbit antibody (Abcam, Cambridge, UK). After washing, the bands were detected by chemiluminescence and imaged with X-ray films. β-actin was used as an endogenous reference for normalization.

### Animal xenografts

Nude mice were purchased from Shanghai SLAC Laboratory Animal Co.,Ltd. (Shanghai, China) and housed at the animal experiment center of Zhengzhou University, where the implantation experiment was performed. All the protocols were previously approved by the Zhengzhou University Animal Ethics Committee. HepG2 cells (1 × 10^6^) were suspended in 200 μl medium, and injected subcutaneously into the flank regions of 48 female athymic nude mice. Two weeks after inoculation, visible subcutaneous tumors were detected, and the tumors were measured approximately 2.5 mm in diameter 3 weeks after inoculation. All animals were randomly divided into 3 groups (8 mice per group): the Model group, the NC group, and the lncRNA-CR594715-silencing group. For the intervention groups, each animal received 30 μl recombinant lentivirus (1 × 10^8^ IFU) twice a week since the second week for 4 weeks, while the model group received the same volume of saline instead. Tumor diameter was measured weekly since the second week, and the data was used to plot the tumor growth curves. The formula for calculating the tumor volume was: V = 1/2 a × b2, a and b are the long and short diameters of the tumor, respectively.

### Statistical analysis

All data are expressed as mean ± SD, and analyzed by one way ANOVA. Least Significant Difference (LSD) was used for multiple comparisons between any two means. *P*-values < 0.05 were considered statistically significant. All statistical analysis was performed using SPSS 13.0 software.

## Results

### Assessment of mRNA and protein levels of CTNNB1 and hsa-miR-142-3p and lncRNA-CR594175 levels through RT-qPCR and Western blotting in adjacent non-cancerous, primary HCC and metastatic tissues

**Fig. 1:**
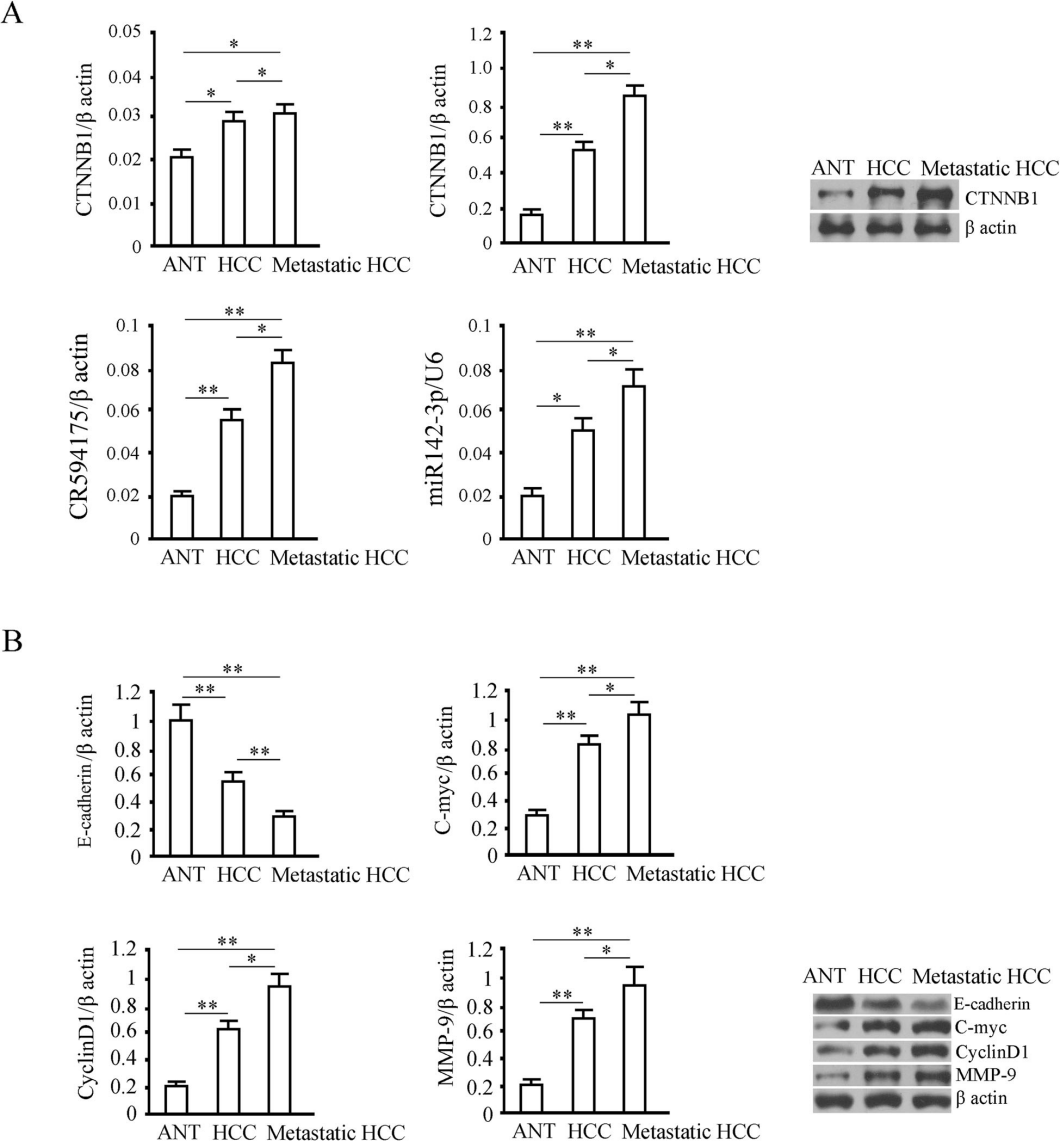
Detection of expression levels of lncRNA-CR594175, hsa-miR142-3p, CTNNB1 and proteins related to Wnt pathway in adjacent normal tissue (ANT), the HCC tumors and their metastasis. **a** The levels of lncRNA-CR594175, hsa-miR142-3p and CTNNB1 mRNA were detected by Real-Time PCR; in the right panel the CTNNB1 protein evaluated by western blotting shows a higher level in metastatic tissues. **b** mRNA and protein levels of E-cadherin (95 kDa), C-myc (56 kDa), CyclinD1 (36 kDa) and MMP-9 (102 kDa) in non-tumor adjacent tissues, HCC and metastatic tissues were detected by Real-Time PCR and western blotting, respectively: representative blots; the optical density of the target band divided by the optical density of the β-actin band; Data are expressed as mean ± SD. *, *p* < 0.05 and **, *p* < 0.01, t-test

The data of CTNNB1 mRNA and protein levels demonstrated that in comparison with adjacent non-cancerous tissues, CTNNB1 protein was increased in HCC and its metastasis (*p* < 0.01), and more in HCC metastasis than in primary HCC (*p* < 0.05); but there were no obvious differences between the mRNA levels in the three groups of tissues (*p* > 0.05). These results suggest that the high expression of CTNNB1 is due to inactivation of post-transcriptional regulation. The levels of lncRNA-CR594175 and hsa-miR142-3p in the adjacent tissues, HCC and its metastasis were positively correlated with CTNNB1 protein levels and was higher in the HCC and their metastasis than that in the adjacent tissues (*p* < 0.05) (Fig. 1a). We also evaluated the expression of downstream functional proteins of Wnt pathway, E-cadherin, C-myc, CyclinD1, and MMP-9, in HCC, in metastasis and adjacent tissue (*p* < 0.01), and we observed that protein levels were significantly higher in HCC metastasis than in primary HCC (p < 0.05). A reverse trend was observed in E-cadherin to the three proteins mentioned above in these tissues (Fig. 1b).

### Effect of lncRNA-CR594175 silencing and CTNNB1 expression via lentiviral strategy on HCC cells

**Fig. 2:**
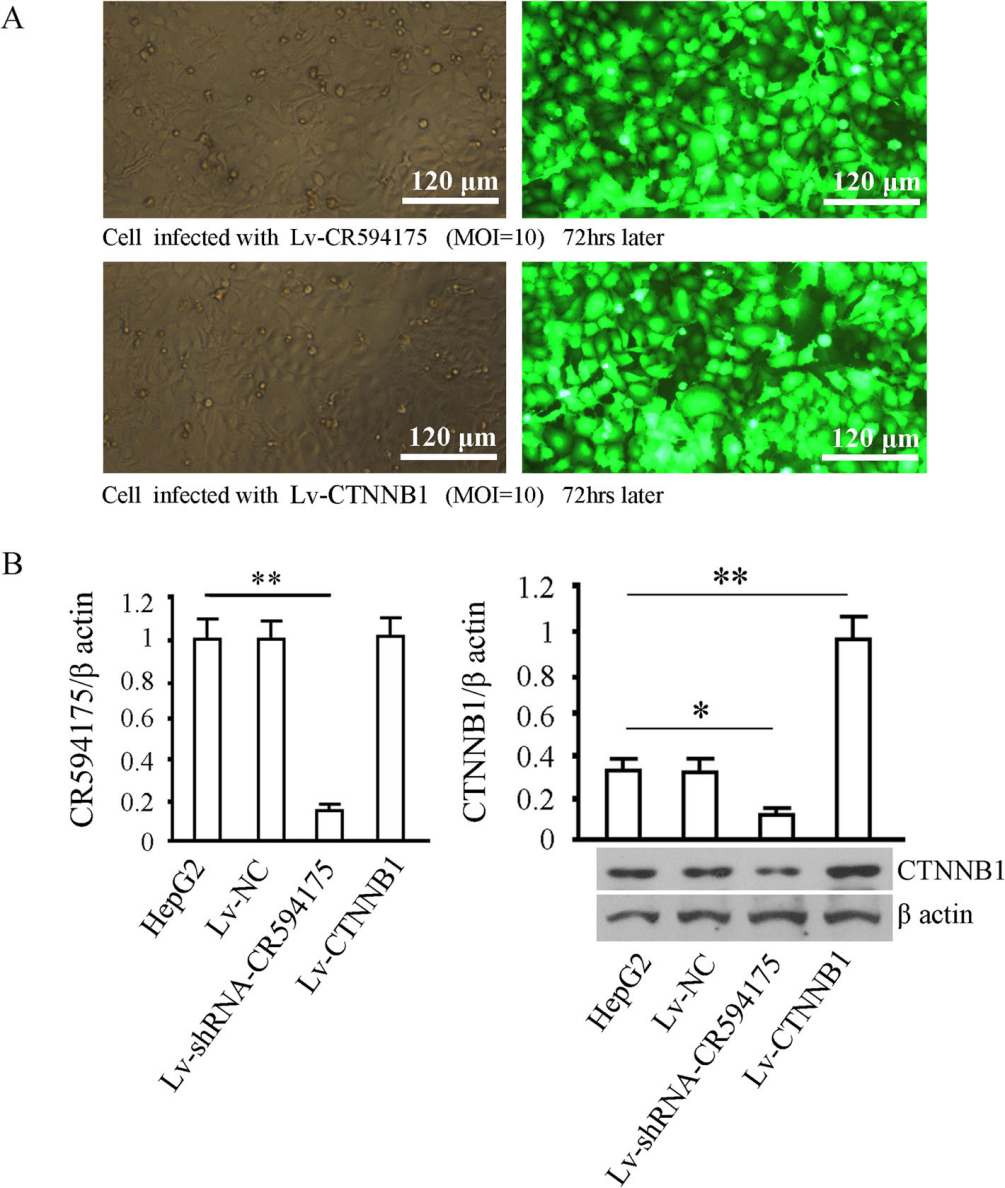
Genetic intervention through a lentiviral approach. **a** GFP expression 72 h after HepG2 were infected with recombinant viruses Lv-shRNA-CR594175 and Lv-CTNNB1. The infection rate was estimated by dividing the number of the cells expressing GFP with the number of all the cells in each view. For statistics, five views were randomly selected, and the mean was calculated; **b** Evaluation of lncRNA-CR594175 and CTNNB1 levels after infection with lentiviruses. For RT-qPCR and western blotting, the β actin was used as internal reference. ** *p* < 0.01, vs cell group. The tests were carried out on three biological triplicates, and data are expressed as the mean ± SD

Recombinant lentiviruses, Lv-NC, Lv-shRNA-CR594175 and Lv-CTNNB1, were used to infect HepG2. GFP (Green fluorescent protein) was detected in most of the cells 72 h after infection, and the proportion of GFP-expressing cells suggested that the gene delivery efficiency was higher than 95% in the HepG2 (Fig. 2a). LncRNA-CR594175 was significantly decreased by Lv-shRNA-CR594175 (*p* < 0.05), and no change in cells infected with Lv-CTNNB1(*p*>0.05) was obsserved; CTNNB1 protein level was significantly increased by Lv-CTNNB1 and decreased by Lv-shRNA-CR594175 (*p* < 0.05) (Fig. 2b). These findings suggest that lncRNA-CR594175 silencing down-regulated CTNNB1 expression in HepG2and that the overexpression of CTNNB1 had no obvious effect on lncRNA-CR594175.

### Luciferase experiments

**Fig. 3:**
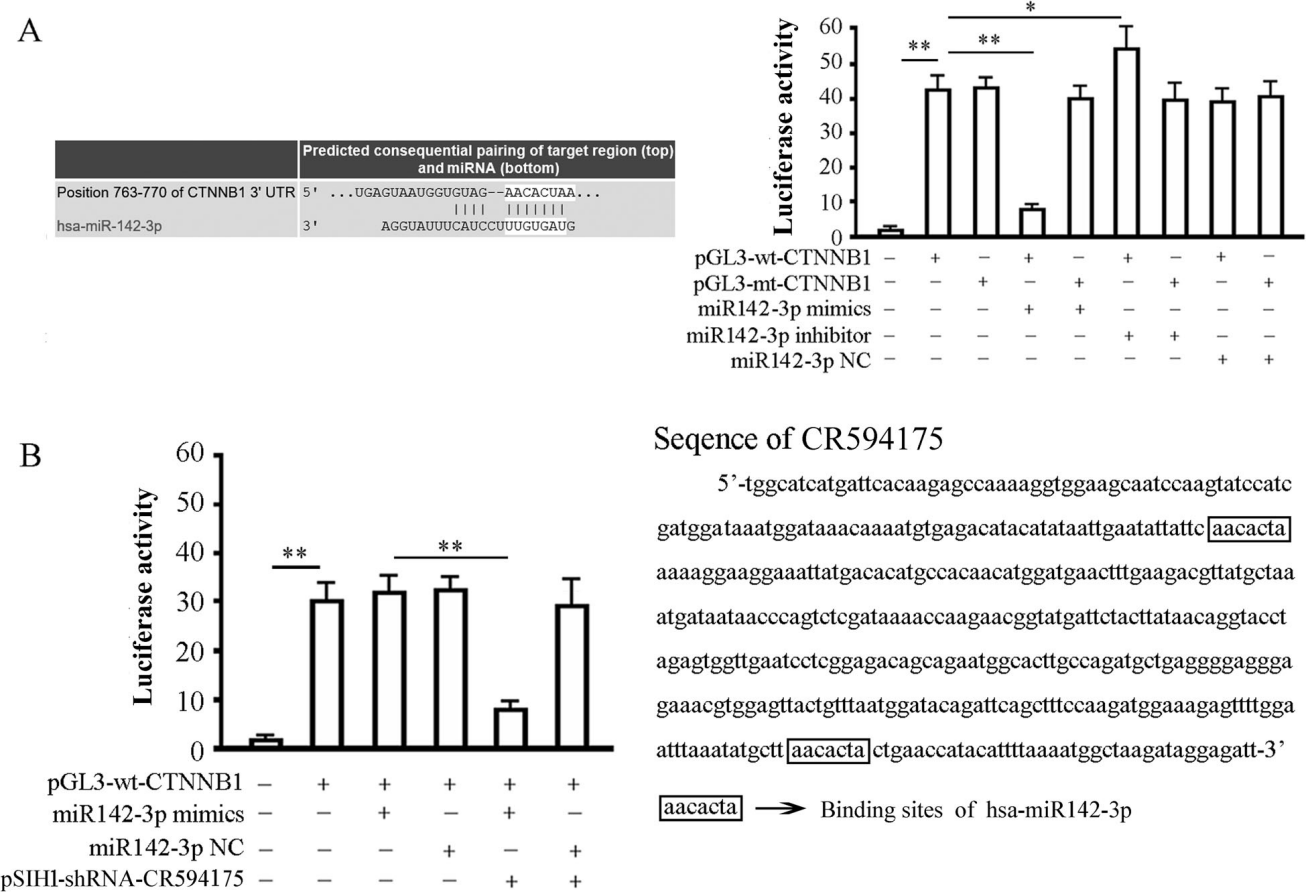
Hsa-miR142-3p binds to CTNNB1 3’UTR, which is interfered by lncRNA-CR594175. **a** 293TN cells were transfected with pGL3-wt-CTNNB1 or pGL3-mt-CTNNB1 in the presence or absence of miR142-3p-mimic or inhibitor and subjected to luciferase activity assay 48 h later. Left, predicted binding site of hsa-miR142-3p in 3′-UTR of CTNNB1; Right, effects of hsa-miR142-3p on the expression of a luciferase cassette encoding the CTNNB1 3′-UTR. The histogram shows the relative firefly luciferase activity for the different experimental groups. *, *p* < 0.05, and **, *p* < 0.01, compared with the group transfected with the same vector but without the miR142-3p mimics or miR142-3p inhibitor. **b** Effect of lncRNA-CR594175 silencing on regulation of CTNNB1 by hsa-miR142-3p in HepG2 cells. Left, HepG2 cells were transfected with the indicated vectors and subjected to luciferase activity assay 48 h later. The histogram shows the relative firefly luciferase activity for the different experimental groups; right, prediction of the binding sites of hsa-miR-142-3p in lncRNA-CR594175. *, *p* < 0.05, compared with the group transfected pGL3-wt-CTNNB1 and miR142-3p-mimics. Data are expressed as mean ± SD of at least three independent experiments

Our bioinformatics analysis identified a 7-base pair hsa-miR-142-3p in the 3′ UTR of CTNNB1 mRNA. We therefore constructed luciferase reporter vectors to verify whether this site represents a valid hsa-miR142-3p target. Reporter vectors containing the wild-type *CTNNB1* 3′-UTR or a variant were generated. The variant vector had the has-miR142-3p target site within the 3′-UTR of *CTNNB1* mutated. Both reporter constructs expressed luciferase at a high level. However, the miR142-3p mimic significantly inhibited luciferase activity in cells transfected with the reporter vector encoding the wild type 3′-UTR (42.15 ± 3.98 vs. 8.07 ± 0.88; *p* < 0.01), while the miR142-3p inhibitor significantly increased luciferase activity in these cells (42.15 ± 3.98 vs. 52.81 ± 9.04; *p* < 0.05) (Fig. 3a). Conversely, in cells transfected with the reporter vector encoding the mutated hsa-miR142-3p target site, neither the miR142-3p mimic nor the miR142-3p inhibitor had any significant effect on luciferase activity (*p* > 0.05). Co-transfection of miR142-3p-NC (non-targeting control) had no effect on the luciferase activity of either of the vectors (*p* > 0.05). These results verified the presence of a hsa-miR142-3p target site in the 3′-UTR of CTNNB1 mRNA and demonstrated that binding of hsa-miR142-3p to this target site down-regulated CTNNB1 expression (Fig. 3a). Interestingly, miR142-3p mimics lost its inhibition on the activity of luciferase expressed by wild-type (wt) luciferase reporter vector in HepG2, and regained the inhibition after lncRNA-CR594175 silencing (Fig. 3b). Taken together, these data suggested that lncRNA-CR594175 silencing could restore the negative regulation of hsa-miR-142-3p on its target gene CTNNB1 in HepG2 cells.

### Effect of lncRNA-CR594175 silencing on proliferation and invasion of HepG2 cells

**Fig. 4:**
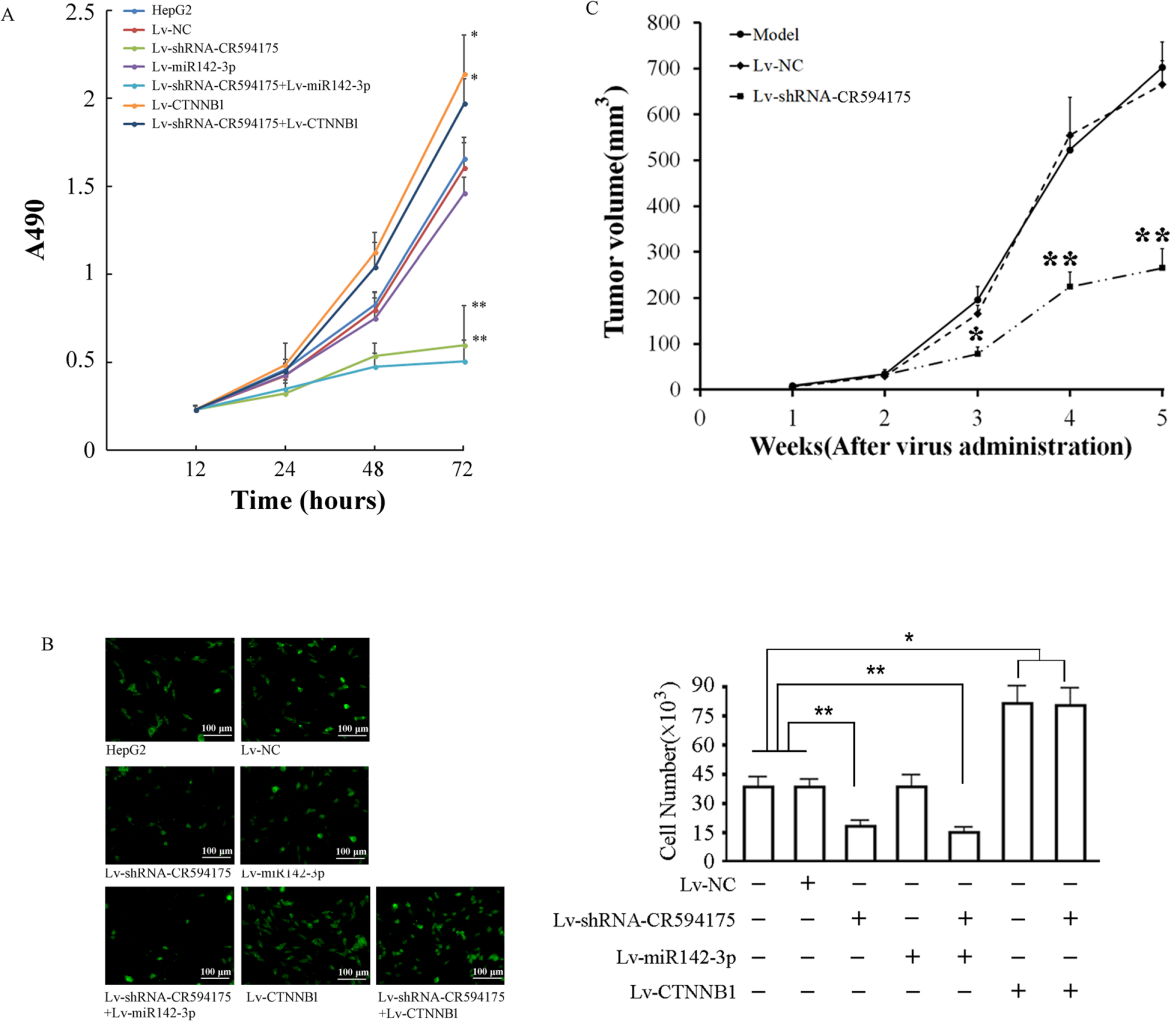
Effects of lncRNA-CR594175 depletion on proliferation and invasion of HCC cells and In vivo tumor suppression. **a** Cell proliferation activity assay. HepG2 were infected with the indicated lentivirus and then seeded to 96-well plates and used for the detection of cell viability at the 0,24,48 and 72 h. The x-coordinate represents proliferation at different time points and the y-coordinate represents the absorbance at 490 nm. **b** Cell invasion assay: HepG2 cells 48 h after infection with the indicated lentiviruses; representative images of cells that seeded into the upper chamber of a transwell and passed through the basement membrane. **c** Growth curves of tumor in vivo*.* The x-coordinate represents the period of virus injection and the y-coordinate represents the tumor volume (mm^3^). The formula for calculating the tumor volume was: V = 0.5 × a × b × b, where a and b are the long and short diameters of the tumor. The number of animals in one group was 12 (*n* = 12). ** *p* < 0.01, *p < 0.05, t-test, data are expressed as the means ± SD

Cell proliferation assay showed that lncRNA-CR594175 silencing inhibited the proliferation of HepG2 cells 72 h after infection (*p* < 0.01,vs. cell group,72 h), while CTNNB1 overexpression promoted the proliferation of HepG2 cells (*p* < 0.05,vs. cell group,72 h), and could reverse the proliferation suppression caused by lncRNA-CR594175 silencing (p < 0.05,vs. lncRNA-CR594175 silencing group,72 h). Overexpression of miR142-3p showed a strong inhibitory activity of proliferation in the case of lncRNA-CR594175 silenced(*p* < 0.01,vs. cell group,72 h) (Fig. 4a). Therefore, it was suggested that lncRNA-CR594175 silencing inhibited cell proliferation by suppressing the expression of CTNNB1 protein. Compared with the control group, the invasion ability of HepG2 in the lncRNA-CR594175 silence group was significantly weakened (*p* < 0.01 vs. control group), significantly enhanced in CTNNB1 overexpression group and lncRNA-CR594175 silence combined CTNNB1 overexpression group (*p* < 0.05 vs. control group), and there was no significant change between the NC group or miR142-3p overexpression group and control group (*p* > 0.05 vs. control group). The invasive ability of HepG2 in the lncRNA-CR594175 silence group combined miR142-3p overexpression group was significantly weakened than that of the control or NC group (*p* < 0.01 vs. control group or NC group), but there was no significant difference from the lncRNA-CR594175 silence group (*p* > 0.05 vs. miR142-3p overexpression group) (Fig. 4b). In vivo experiment showed that 4 consecutive weeks of treatment with Lv-shRNA-CR594175 significantly reduced the tumor volume. After administration for five weeks, the tumor volume of the model group was 701.21 ± 54.13 mm^3^, the NC control group was 672.34 ± 49.06 mm^3^ and the lncRNA-CR594175 silencing group was 212.31 ± 57.71 mm^3^. The tumor inhibition rates in the NC group and lncRNA-CR594175 silenced group were 4.12 and 69.73%, respectively, with a statistically significant difference between the lncRNA-CR594175 silenced group and the other two groups (*p* < 0.01, vs. Model group or NC group) (Fig. 4c).

### Effect of lncRNA-CR594175 silencing on expression of downstream functional protein of Wnt pathway

**Fig. 5:**
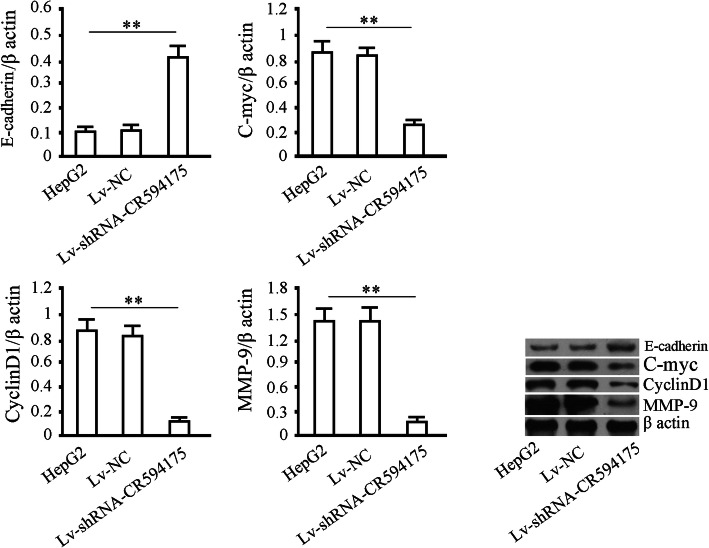
Effects of CR594175 lncRNA-CR594175 silencing on E-cadherin, C-myc, CyclinD1 and MMP-9. HepG2 were infected with Lv-NC (negative control) or with Lv-shRNA-CR594175 and 72 h later were subjected to western blotting to test protein levels downstream of the WNT pathway. β actin (43 kDa) was used as the loading control. Data are representative of at least three independent experiments. ** *p* < 0.01, *p < 0.05.t-test

We assessed E-cadherin, C-myc, CyclinD1 and MMP-9 in the lncRNA-CR594175 silenced HepG2 cells. The results showed that C-myc, CyclinD1 and MMP-9 were decreased and E-cadherin was increased by lncRNA-CR594175 silencing significantly (p<0.01) but no change in protein levels was observable after treatment with Lv-NC (p>0.05) (Fig. 5). The results indicated that lncRNA-CR594175 silencing could regulate the classic Wnt pathway by reducing CTNNB1 protein levels and the other Wnt pathway activation related proteins modulating cell proliferation and invasiveness (Fig. 5).

## Discussion

The invasion and metastasis of cancer refers to cancer cells that break away from the primary focus of the tumor and transfer to the neighbor where they proliferate into cancer of the same nature [13]. This process depends on the interaction between cancer cells and tumor microenvironment promoting their survival, growth, and angiogenesis, as well as invasion and metastasis [14]; therefore, inhibiting of proliferation and invasion of tumor cells is the key factor to inhibit tumor metastasis.

As an important type of regulators, lncRNAs exert their functions through a variety of ways. Although they were firstly regarded as by-products by RNA polymerase II, or transcriptional noise, recent studies have shown that lncRNAs are associated with multiple biological processes such as chromosome silencing, chromatin modification and transcriptional regulation [15, 16]. The proportion of lncRNAs in the total transcripts of genome is far larger than that of encoding RNAs. And lncRNAs play crucial roles in the regulatory network by their interaction with DNA, RNA and proteins. In addition to gene expression regulation, lncRNAs are closely related to evolution of species, embryonic development, metabolism and tumorigenesis. The evidence on involvement of lncRNAs in diseases including cancers will provide basis and target for diagnosis and treatment of diseases. Sun Shu-han et al. have found lncRNA-Dreh can inhibit hepatocellular carcinoma metastasis [17].

We screened for differential lncRNAs in several pairs of selected HCC and adjacent tissue by using lncRNA chips. The reason why lncRNA-CR594175 caught our attention was that its expression was not only increased in HCC than in adjacent non-cancerous tissues, but also was increased more in HCC metastases than in primary HCC, indicating that lncRNA-CR594175 would be associated with the process of HCC occurrence and metastasis. We knocked down lncRNA-CR594175 in HepG2 cells, and found that the proliferation and invasion were reduced, as well as downstream proteins of Wnt pathway cMyc, CyclinD1 and MMP-9, through Wnt pathway while E-cadherin shows an opposite behavior. So we believed that high lncRNA-CR594175 levels may contribute to metastases formation and tumor progression by regulating proteins downstream of Wnt pathway. Wnt signaling pathway is one of the key signaling pathways for cell proliferation and differentiation. To figure out how lncRNA-CR594175 promotes Wnt pathway in HCC, we analyzed key proteins involved in invasion and migration in HCC cells with lncRNA-CR594175 silencing and control cells, and found that the expression of CTNNB1 was consistent with lncRNA-CR594175, which was confirmed in primary HCC, metastatic HCC and adjacent tissue. Interestingly, our RIP (RNA Binding Protein immunoprecipitation) experiment showed that lncRNA-CR594175 did not bind to CTNNB1 directly (Data not shown). Next we quantified the transcription and protein levels of CTNNB1 in HCC with lncRNA-CR594175 silencing and found that CTNNB1 expression was abnormal at the post-transcription level, suggesting a mechanism of regulation of CTNNB1 expression following the high expression of lncRNA-CR594175. As a typical post-transcription regulating factor, miRNA naturally became our pointcut to investigate the relation between lncRNA-CR594175 and CTNNB1. Bioinformatics suggests that there is a 7 base-pair seeding region of hsa-miR142-3p on CTNNB1’s 3’UTR and 2 seeding regions on the 600 base-pair lncRNA-CR594175. As a result, we speculated that elevated lncRNA-CR594175 bound to hsa-miR142-3p as miRNA sponge and disabled the negative regulation of CTNNB1 by hsa-miR142-3p, so CTNNB1 expression was increased and resulted in proliferation and invasion of HCC cells.

The interaction between lncRNAs and miRNAs has an important influence on the onset and development of cancer [18]. MiRNAs are able to regulate lncRNAs in a targeted way: a study has shown that miR-21 targets lncRNA GAS5 in addition to protein coding genes [19]. LncRNAs can also affect the onset and development of cancer by regulating expression of miRNAs [20]. According to existing studies, lncRNAs regulate miRNAs through three ways: (1) combining competitively to 3′-UTR of mRNAs so inhibiting negative regulation by miRNAs. Faghihi et al., for example, found that an anti-sense RNA can bind to BACE1 mRNA, competitively inhibiting the negative regulation of BACE1 by miRNA [21]; (2) to regulate target genes by forming pre-miRNAs after RNA splicing and producing specific miRNAs [22, 23]; and (3) to act as endogenous miRNA sponge to suppress miRNA function, so as to affect malignant biological behavior of cancer cells [24]. The most important finding of this study is that lncRNA-CR594175 silencing could restore the negative regulation of CTNNB1 by hsa-miR142-3p to inhibit cancer, directly based on following facts: (1) hsa-miR142-3p negatively regulated CTNNB1 by binding to its 3’UTR, which was found in HCC with lncRNA-CR594175 silencing but not those with high lncRNA-CR594175 expression levels; (2) LncRNA-CR594175 silencing inhibited proliferation and invasion of HCC cells, which was reversed by overexpression of CTNNB1; (3) Overexpression of hsa-miR142-3p had no observable effect on proliferation and invasion of HCC cells, but inhibited proliferation and invasion of HCC cells when lncRNA-CR594175 was depleted. Considering that CTNNB1 overexpressed by the lentiviral system has no wild 3’UTR, it would not be affected by miRNA. So we think that there is a lncRNA-CR594175/hsa-miR-142-3p/CTNNB1 axis regulating metastasis formation in HCC.

## Conclusion

The study demonstrates that lncRNA-CR594175 plays a key role in the process of HCC metastasis, and offers a possible explanation about why hsa-miR142-3p loses its basic function of resisting HCC tumors. In the long run, lncRNAs will not only be a direct target for gene therapy but it can also be used together with miRNAs for a better effect.

## Data Availability

All data generated or analyzed during this study are included in this published article [and its supplementary information files].

## Abbreviations

HCC: Hepatocellular carcinoma
CTNNB1: Catenin, beta-1
UTR: Untranslated Regions
FBS: Fetal bovine serum
DMEM: Dulbecco minimum essential medium
NC: Negative control
bp: base-pair
LSD: Least Significant Difference
GFP: Green fluorescent protein
